# HyperTransFusion: a hypernetwork transformer with black winged kite optimization for multimodal early Alzheimer's disease diagnosis

**DOI:** 10.3389/fdgth.2026.1874626

**Published:** 2026-08-12

**Authors:** S. Sabari Vasan, P. Jayalakshmi

**Affiliations:** School of Computer Science Engineering and Information Systems, Vellore Institute of Technology, Vellore, India

**Keywords:** adaptive fusion, Alzheimer disease, disease detection, multimodal fusion, vision transformer

## Abstract

**Introduction:**

Early detection of Alzheimer's disease (AD) is critical for timely intervention and effective disease management. Existing diagnostic systems are often limited by unimodal data or static fusion strategies that fail to capture complex interactions between neuroimaging and cognitive biomarkers.

**Methods:**

A HyperTransFusion framework was proposed for AD classification using MRI scans and cognitive assessment scores from the ADNI dataset. The framework integrates a Hypernetwork-based Transformer for adaptive multimodal fusion, a Vision Transformer (ViT) for MRI feature extraction, dense embedding layers for cognitive features, and a BWKO-SA optimization strategy combining Black-Winged Kite Optimization and Simulated Annealing for hyperparameter tuning. Performance was evaluated using five-fold patient-wise cross-validation.

**Results:**

The proposed framework achieved a macro AUC of 0.9311 ± 0.039 and an overall accuracy of 0.8624 (95% CI: 0.7691-0.9558), demonstrating robust generalization across unseen subjects. Ablation studies confirmed the contribution of the Hypernetwork and BWKO-SA modules. Slice-level evaluation achieved a peak accuracy of 99.1%, reported only as a supplementary indicator of feature separability.

**Discussion:**

The proposed HyperTransFusion framework enables adaptive multimodal integration and improves the accuracy of early AD classification. Further validation using independent multi-center cohorts is required before clinical translation.

## 1 Introduction

Alzheimer's is a major contributor to dementia and gradually affects cognitive and memory functions. It may prove difficult to detect this condition early owing to several factors such as the gradual nature of the condition, disease variability, slow progression, and the subjectivity of clinical assessments (1).

AD typically progresses from cognitively normal (CN) status through Mild Cognitive Impairment before reaching the advanced stage, making early identification of disease progression clinically important. Advancements in neuroimaging technologies and deep learning methodologies have enabled the development of multimodal frameworks that leverage multiple biomarkers to improve AD assessment accuracy. A deep metric-learning-based multimodal model demonstrated that lesion-specific feature extraction and cross-modal relationship learning can enhance diagnostic performance (2). Similarly, better performance in diagnosis was attained through the use of a feature integration approach whereby redundant representations were reduced, but complementary information from Magnetic Resonance Imaging (MRI) and Fluorodeoxyglucose Positron Emission Tomography (FDG-PET) imaging is emphasized (3). Additionally, multimodal learning improved through hierarchical aggregation and attention-driven interactions among multiple representations, resulting in competitive performance on the Alzheimer's Disease Neuroimaging Initiative (ADNI) dataset (4).

However, despite these developments, a meta-analysis of 47 studies revealed high variability in multimodal fusion approaches and emphasized the importance of developing a better clinical classification method for diagnosing AD (5). Moreover, a systematic review of the machine learning and deep learning approaches used for predicting the development of AD in MCI cases revealed major challenges associated with data integration, robust representation learning, and generalization within heterogeneous biomarker datasets (6). Apart from applications in medical imaging, recent studies revealed that adaptive parameter generation techniques can enhance the process of representation learning in complex learning settings (7). Nevertheless, effectively capturing the intricate relationships among heterogeneous neuroimaging and cognitive biomarkers remains a tough task. Existing multimodal approaches often exhibit limited adaptability to inter-subject variations and may not fully exploit complementary information from diverse clinical indicators. Therefore, developing a robust multimodal framework that improves feature interactions, representation learning, and diagnostic reliability remains an important research direction in AD diagnosis.

In order to overcome the constraints posed by currently available multimodal Alzheimer's Disease (AD) diagnosis models, this paper presents a HyperTransFusion framework, which includes MRI data and cognitive biomarkers for AD detection. The proposed framework makes use of adaptive multimodal learning and optimization techniques to improve effective feature interactions, learning representation and reliability of the framework.

### 1.1 Key contributions

- A novel HyperTransFusion framework is proposed for multimodal AD diagnosis by integrating MRI images and cognitive assessment scores within a unified architecture.
- A Hypernetwork-based Transformer is developed to dynamically generate modality-aware fusion weights, enabling adaptive interaction between neuroimaging and cognitive features.
- A hybrid Black-Winged Kite Optimization–Simulated Annealing (BWKO-SA) algorithm is introduced to optimize model hyperparameters and improve convergence stability.
- Extensive experiments on the ADNI dataset, including ablation studies and five-fold cross-validation, evaluate the effectiveness, robustness, and generalisation capability.

### 1.2 Novelty

The proposed work contributes to the development of a HyperTransFusion framework that integrates a hypernetwork-based transformer with multimodal MRI and cognitive biomarkers for Alzheimer's disease detection. Unlike traditional methods, which use static fusion schemes, our approach learns modality-specific fusion parameters to capture subject-specific interactions among heterogeneous biomarkers. Furthermore, the integration of the hybrid Black-Winged Kite Optimization–Simulated Annealing (BWKO-SA) algorithm enhances hyperparameter optimization and convergence stability, resulting in more robust multimodal representation learning and improved diagnostic performance.

## 2 Literature review

Numerous AI-driven frameworks, including CNNs, Transformer-based networks, and hybrid architectures, have been utilised to predict MCI progression as well as enhance AD diagnosis. Murugan et al. (8) used a CNN model with MRI images as input data and obtained impressive results, but combining manual as well as CNN-based features was inadequate for discriminating between different AD stages. To address these problems, Altwijri et al. (9) proposed a deep learning-based diagnostic MRI model with a 99.3% success rate. However, the effectiveness of a model is limited by its small database and limited image-processing capabilities.

Sharma et al. (10) designed a hybrid framework consisting of two stages, in which deep features were initially extracted using a dual DenseNet, followed by the application of a voting classifier in combination with a set of traditional machine learning classifiers. Even though this framework provided promising results, its complexity made it more vulnerable to overfitting and less scalable, which inspired the use of Transformer models.

According to Yan et al. (11), the Hybrid-RViT architecture was designed to use both ResNet-50 and ViT, thereby reducing the differences between CNN and Transformer models. As for the AD stage classification task, hybrid models achieved 97% training accuracy and 95% testing accuracy. An attempt to move further toward 3D models is represented by the MIMD-3DVT model from Castro et al. (12), which fused consecutive 3D MRI slices and achieved 97.14% accuracy despite using only a few patients and the difficulty of training the 3D ViT network. Another combination, the VGG-TSwinformer model by Hu et al. (13), was used to predict MCI progression from longitudinal sMRI scans and achieved 77.2% accuracy and an AUC of 0.8153, but suffered from fusion difficulties across dimensions.

The design of transformer architectures and hybrid deep learning models has attracted considerable interest in recent research related to AD detection. For example, Aslan et al. (14) introduced a Multi-Axis Vision Transformer (MaxViT) architecture for four-stage AD classification based on MRI images; however, the employment of augmented data and performance on the Kaggle dataset negatively impacted its generalizability in practice. Among other approaches compared by Aslan et al. (15), there were classical machine learning classifiers such as K-Nearest Neighbour (KNN), Support Vector Machine (SVM), AdaBoost, and Logistic Regression. Despite encouraging results obtained by these models, the authors mentioned the need for their improvements via proper feature selection and hyperparameter optimization. In addition, Aslan et al. (16) suggested the VGG19-U-Net model for MRI segmentation of AD patients; still, the applicability of this model to different data was not guaranteed. Alpsalaz et al. (17) developed the fuzzy ensemble system, which comprised InceptionV3, MobileNetV2, and Inception-ResNetV2 models and gave 94% accuracy and 0.99 AUC but entailed heavy computation costs and lack of multimodality adaptiveness.

Nevertheless, predicting a progression of MCI patients into AD is still a complicated process. Abdelhameed et al. (18) developed a prediction model based on BiGRU with AUC-ROC scores of 0.833 as well as 0.8153. However, the prediction model is highly dependent on medical record and is adversely affected by a negative effect of missing medical information. On the other hand, Ma et al. (19) proposed a Transformer model based on CHARLS dataset, with an accuracy of more than 90% and an MAE of 3.5. In addition, Ghadami et al. (20) proposed GLCM along with HHO as well as deep learning models based on MLP and LSTM networks. This combination of the two models not only improved the predictive power but also added complexity to computation. Furthermore, Almubark et al. (21) used deep learning models for cognitive assessment with 90% sensitivity along with specificity. Lastly, Wang et al. (22) introduced a hybrid machine learning model which needs re-training when new information is obtained, thereby limiting its scalability in clinical practice.

Recent works have increasingly been concentrating on Transformer-based multimodal fusion methods for enhancing the diagnostics of Alzheimer's disease via integrating clinical data of different types. Tang et al. (23) presented an enhanced Transformer-based method for fusing MRI and PET scans for the better diagnostics but still limited by imaging techniques only and not including cognitive assessment data. In turn, Yu et al. (24) developed a unified multimodal Transformer model based on MRI, clinical, and genetic data, which led to improved representation learning for different modalities, yet the method required static parameter optimization. In order to resolve this issue, Duenias et al. (25) introduced HyperFusion, a Hypernetwork-based multimodal framework that dynamically generated image-processing parameters from clinical data, achieving superior classification performance at the expense of increased computational complexity during training.

Further improvements considered more adaptive multimodal architectures. Kadri et al. (26) have developed a hybrid transformer and adaptive MLP-mixer model that utilized MRI, PET, DTI, and fMRI data in order to provide accurate diagnosis, but it consisted of several complicated networks. Chen et al. (27) have created a multimodal CNN-Transformer architecture, which helped to better recognize Alzheimer's disease using complementary features learning, however, its strategy of feature fusion was still static. Wang et al. (28) have suggested a multimodal hypergraph learning model that united imaging and genetic data for high-order modality interactions detection, but the complexity of the hypergraph model raised.

Recent studies further demonstrated that a longitudinal multimodal fusion model (29) showed better classification of Alzheimer's disease through the use of temporal information in imaging but did not include cognitive assessment information in feature extraction. In addition, in a meta-analysis study by Guo et al. (30), Transformer multimodal fusion models showed that multimodal systems outperformed single-modal methods. Nevertheless, existing multimodal fusion methods continue to face challenges related to adaptive feature interaction, computational efficiency, and cross-cohort generalization. These limitations motivate the proposed HyperTransFusion framework, which employs Hypernetwork-guided adaptive fusion of MRI and cognitive features together with BWKO-SA optimization to improve multimodal feature learning, optimization stability, and Alzheimer's disease classification performance.

### 2.1 Research gaps

Although recent studies have improved Alzheimer's disease diagnosis using Transformer-based and multimodal learning approaches, several limitations remain. Most existing methods rely on static multimodal fusion, insufficiently exploit the complementary information between MRI and cognitive assessments, or introduce high computational complexity. Moreover, Hypernetwork-based and adaptive fusion methods still face challenges related to optimization efficiency, scalability, and generalization across heterogeneous clinical data. Therefore, an adaptive and computationally efficient multimodal framework capable of dynamically integrating MRI and cognitive features while improving optimization and classification performance remains necessary.

## 3 Dataset

ADNI is a massive and multi-disciplinary research which is intended to study the progression of AD by conducting cognitive tests, blood tests, cerebrospinal fluid (CSF) tests, and neuroimaging through MRI/PET scans (31). A total number of 10,000 MRI samples was taken to test an effectiveness of this study, with 80% (8,000 samples) as training data and 20% (2,000 samples) as testing data, as shown in Table 1 below. This data set consisted of three types: CN, MCI, and AD, each with a nearly balanced number of samples. Multimodal inputs include MRI images and cognitive assessment scores, namely the Mini-Mental State Examination (MMSE), Geriatric Depression Scale (GDS), Global Clinical Dementia Rating (Global CDR), Clinical Dementia Rating (CDR), and Functional Activities Questionnaire (FAQ), which are used for model training as well as evaluation. The ADNI dataset consists of 102 unique subjects, from which MRI slices were used as individual input samples for deep learning training. Each subject contributes multiple axial slices, and each slice is assigned the corresponding subject-level diagnostic label (CN, MCI, or AD). The proposed method yields 10,000 labelled MRI scans that are employed to train the model, which guarantees the availability of enough data volume for deep learning while maintaining diagnostic consistency on the subject level. Although the number of MRI slices contributed by each subject varied, no upper-bound truncation, duplication, or sample weighting strategy was applied. Instead, patient-level StratifiedGroupKFold partitioning was adopted to ensure that all MRI slices belonging to the same subject remained within a single fold. Consequently, subjects contributing a larger number of slices did not appear simultaneously in both the training and testing folds, thereby preventing data leakage. This subject-wise partitioning provides an unbiased estimate of the proposed model's generalization performance despite the variation in the number of slices contributed by individual subjects. Moreover, class weights were calculated independently for each training fold through the use of the compute_class_weight() method available in the scikit-learn package and then included in the loss function during model training to reduce the influence of unequal class distributions and improve balanced learning across diagnostic categories.

**Table 1:** Description of ADNI used in proposed.

Category	Parameter	Description
Dataset Composition	Total number of subjects	102 unique participants
	Total MRI samples	10,000 MRI slices
	Training set	8,000 slices (80%)
	Testing set	2,000 slices (20%)
	Data storage structure	MultiModal/ADNI/011_S_XXXXX/Axial_PD-T2_TSE/YYYY-MM-DD_…
Overall Class Distribution	CN	3,323 samples (33.23%)
	MCI	
	AD	
	Subject distribution	37 CN, 26 MCI, and 39 AD subjects
Training Set Distribution	CN	2,638 samples (32.98%)
	MCI	2,685 samples (33.56%)
	AD	2,677 samples (33.46%)
Testing Set Distribution	CN	685 samples (34.25%)
	MCI	659 samples (32.95%)
	AD	656 samples (32.80%)
Participant Characteristics	Age range	55–90 years
	Mean age	Approximately 75 years
	Sex ratio	Approximately equal distribution of male and female participants
	Educational attainment	Mean education level of 14–16 years
	MMSE	CN: 24–30, MCI: 24–30, AD: 20–26
	CDR	CN: 0, MCI: 0.5, AD: 0.5–1.0
	Global CDR score	CN: 0, MCI: 0.5, AD: 0.5–1.0
	GDS	Score < 6 across all groups
	FAQ	Available for all participants; normal in CN and impaired in MCI/AD groups
	Ethnic composition	Predominantly White (>90%)
MRI Slice Distribution	Minimum number of slices per subject	10 slices
	Maximum number of slices per subject	482 slices
	Average slices per subject	98.0 slices
	Median slices per subject	72.0 slices
	Standard deviation	81.5 slices
	Total MRI slices in dataset	10,000 slices
MRI Protocol	Data repository	Alzheimer’s Disease Neuroimaging Initiative (ADNI) database
	Magnetic field strength	Scans acquired using 1.5T systems as primary setup and 3T scanners in ADNI-2/ADNI-3 cohorts
	T1-weighted acquisition	3D MP-RAGE / IR-FSPGR sequences obtained in sagittal orientation
	T2-weighted acquisition	Axial Proton Density–T2 Turbo Spin Echo (PD-T2 TSE) imaging protocol
	Slice thickness	1.5–2.0 mm for T1-weighted images and 3–4 mm for T2-weighted images
Participant Criteria	Age specification	Subjects aged between 55 and 90 years
	Cognitive assessment (MMSE)	CN: 24–30, MCI: 24–30, AD: 20–26
	Clinical staging (CDR)	CN: 0, MCI: 0.5, AD: 0.5–1.0

## 4 Proposed system

**Figure 1:**
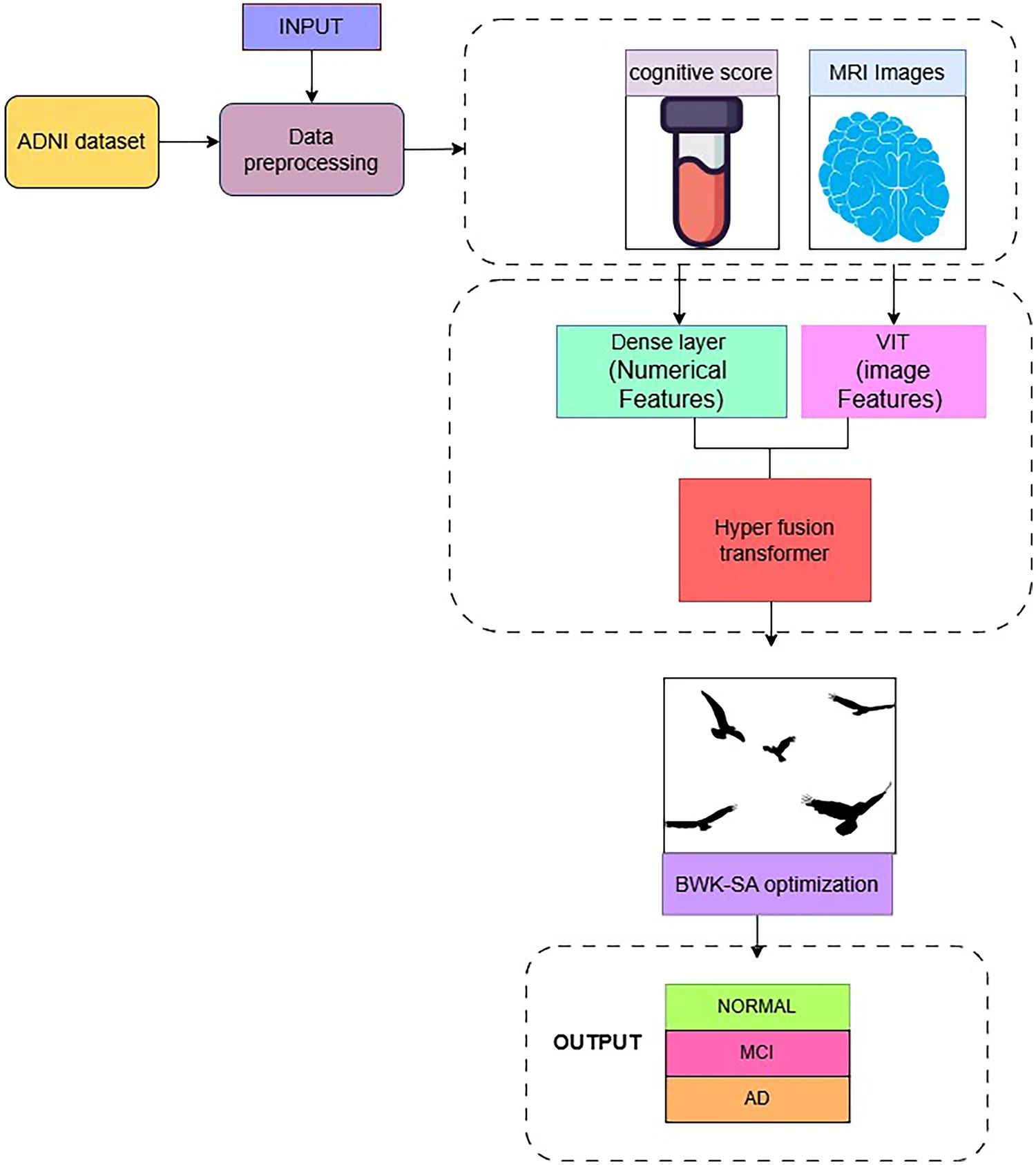
Proposed flow diagram.

HyperTransFusion architecture employs a Hypernetwork Transformer that incorporates ViT to acquire visual features from MRI images, as well as dense layers to extract information related to cognitive assessment tests. Adaptive fusion of these features is done by Hypernetworks, and the BWKO-SA technique of optimization is applied for parameter tuning and ensuring convergence stability. Figure 1 shows an outline of the whole framework.

### 4.1 Preprocessing

The HyperTransFusion method employs a complete preprocessing pipeline to improve input quality that ensures effective feature learning and promotes better generalization. To begin with, cognitive score values are converted to numeric values, after which missing values are appropriately imputed. Specifically, cognitive scores with missing values that exceed a threshold are excluded, while the remaining are filled using mean imputation from the training set.

In this case, the cognitive scores are normalized using standard scaler normalization such that their mean becomes zero and the variance becomes one. The formula for calculating the normalized values is provided below in Equation 1:

$$\;Z=\frac{x-\mu }{\sigma }$$ (1)

where *x* represents individual cognition score, μ is the mean score, whereas refers to standard deviation of samples (32), which are derived solely from a training sample set to prevent information leakage.

To increase model reliability and reduce overfitting, the multiple inputs (both MRI slices and cognition) are randomly shuffled before each training cycle. Moreover, all the images are resized to 224   ×   224 through interpolation to satisfy the requirements of ViT model. This provides consistency in terms of image resizing, data inclusion, and modalities fusion during training.

### 4.2 Multimodal feature representation

**Figure 2:**
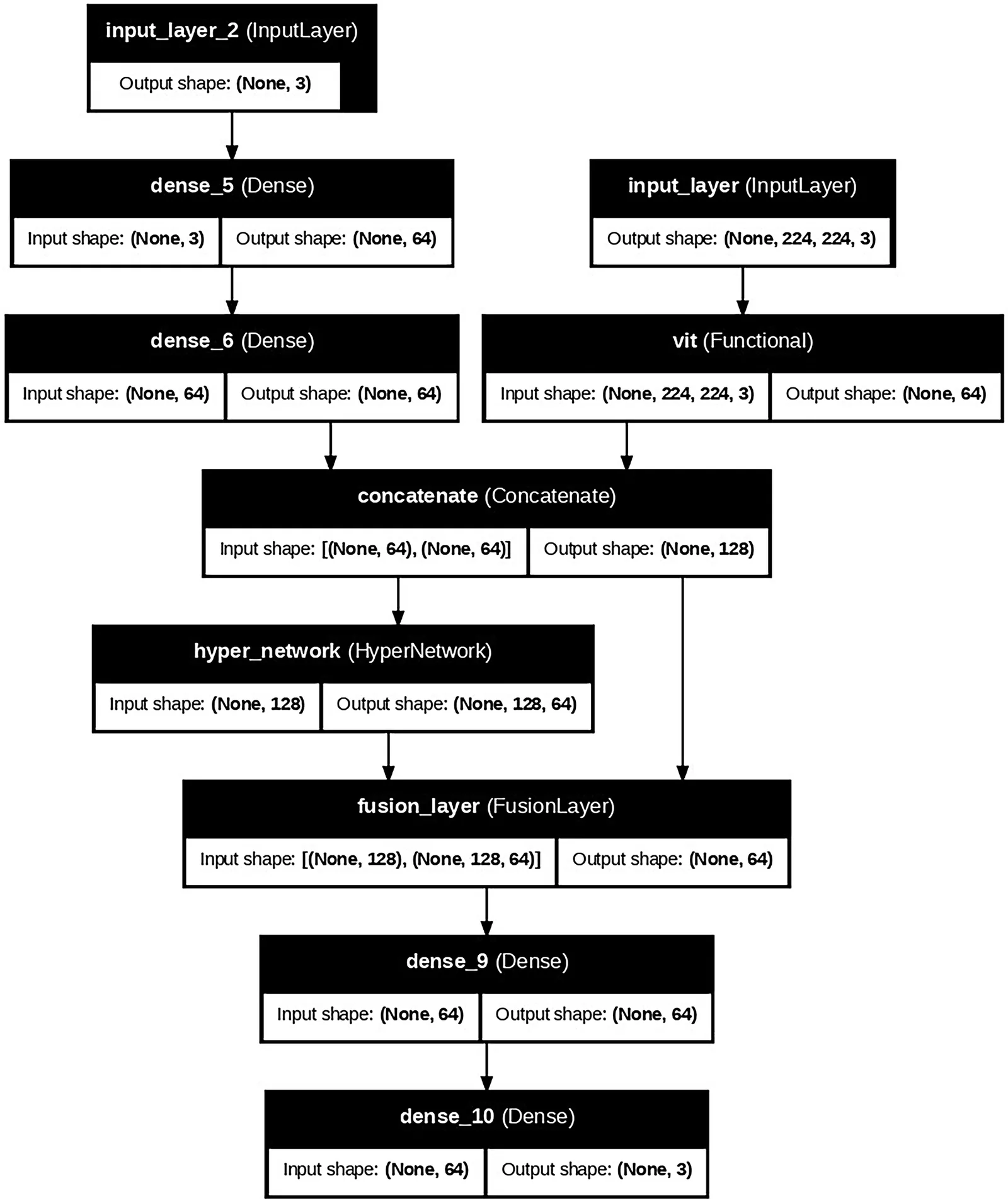
Hypernetwork transformer model.

Following the embedding phase, feature representations are created independently for both cognitive and imaging modalities. In the case of cognitive data, a dense layer is used to create feature representations, as well as for MRI images, ViT is utilized to obtain this based on visual features. On a dense layer, the three-dimensional input is mapped to a 64-dimensional space using a neural network architecture. This helps a model learn the statistics of the data features. The resulting numerical feature vector is expressed in Equation 2.

y=f(Wx+b) (2)

f(⋅) is used to represent activation function applied to the modified input, with *W* being a trainable weight matrix and *b* the bias vector. As illustrated in Figure 2, an additional dense layer is applied to strengthen a representation capability of the cognitive features.

Simultaneously, each axial MRI slice is passed through a pre-trained ViT employing a patch size of 16. The input image is partitioned into non-overlapping 16   ×   16 patches, which are subsequently projected as 64-dimensional feature embedding space. To preserve spatial relationships among image regions, positional encodings are incorporated into an embedded patch representation. The ViT then processes these through multiple Transformer encoder blocks comprising Multi-Head Self-Attention (MHSA) and Feed-Forward Network (FFN) modules, enabling effective learning of discriminative visual representations for subsequent multimodal fusion.

Dependencies between different contexts in visual representations are captured using the MHSA method (33), which is defined mathematically in Equations 3, 4.

$$Multihead\;(Q,K,V)=Concat(\;head_{1},\ldots head_{h})W_{0}$$ (3)

$$\;\;\;\;\;\;\;\;\;\;\;\;\;\;\;\;head_{i}=Multihead\;(QW_{i}^{Q},KW_{i}^{K},VW_{i}^{V})\;\;\;\;\;\;\;\;\;\;\;\;\;\;\;\;\;\;\;\;\;\;\;\;\;\;\;\;\;\;\;\;\;\;\;\;\;\;\;\;\;\;$$ (4)

According to this approach, the attention operation is conducted using a representation for query (Q), key (K), and value (V). In this case, the learnable projection matrices $W_{i}^{Q},W_{i}^{K},W_{i}^{V}$ are used to form vectors. The formed cognitive representations are projected into latent space that is suitable for visual representations obtained from Vision Transformer (ViT) model so that all the feature dimensions would be equal across modalities. Then, the cognitive and MRI feature embeddings are combined using a Hypernetwork-based Transformer that dynamically generates adaptive weight parameters based on the learned multimodal representations. The process of fusion is described in Equation 5:

$$W(z)\;=Fusion(E_{Cog}(c),E_{MRI}(m))$$ (5)

In Equation 5, $E_{Cog}(c)\;$ denotes the feature embedding extracted from the cognitive assessment data, while $E_{MRI}(m)$ represents the feature embedding obtained from the MRI modality. The function Fusion(⋅) performs Hypernetwork-based multimodal feature integration to capture complementary information from both modalities. The resulting fused representation, W(z), serves as the unified feature vector that is subsequently forwarded to the classification network for Alzheimer's disease prediction. The adaptive weight generation mechanism is formulated in Equation 6

$$W_{h}(z)=\left(\begin{array}{c} d_{0}(Z)W_{0}\\ \vdots \\ d_{Nh}(Z)W_{nh} \end{array}\right)$$ (6)

In Equation 6, the notation $d_{0}(Z)$ represents the adaptive control coefficient generated by the Hypernetwork, while $W_{0}\;$ denotes the transformation matrix corresponding to an attention head. The generated adaptive weight matrix $W_{h}(z)$ is subsequently applied during the multimodal fusion process to refine the fused feature representation W(z). This dynamic parameterization enables the Hypernetwork to adapt its attention weights according to the interactions between the cognitive and MRI feature representations, thereby facilitating effective multimodal feature integration (34).

After the fusion process, the fused feature representation is passed through a fully connected classification layer with a SoftMax activation function to predict the diagnostic class (CN, MCI, or AD).

The classification output probabilities are computed using Equation 7.

$$\hat{y}=SoftMax(W_{c}W(z)+b_{c})$$ (7)

where $W_{c}$ denotes the weight matrix of the fully connected classification layer, $b_{c}$ represents the corresponding bias vector, W(z) is the fused multimodal feature representation, and $\hat{y}\;$ denotes the predicted probability distribution over the three diagnostic classes.

The network is trained using the Sparse Categorical Cross-Entropy loss function, which is defined as

$$L=-\frac{1}{N}\sum_{i=1}^{N}log({\hat{y_{i,}}}_{y_{i}})$$ (8)

where N denotes the total number of training samples, $y_{i}$ is the ground-truth class label of the $i^{th}$ sample, and $\hat{y}$ represents the predicted probability corresponding to the true class label. The loss function minimizes the discrepancy between the predicted class probabilities and the ground-truth labels during model training. The BWKO-SA optimization algorithm minimizes the loss function defined in Equation 8 by iteratively updating the HyperTransFusion network parameters, thereby optimizing the multimodal fusion model for accurate Alzheimer's disease classification.

### 4.3 Adaptive model optimization using BWKO-SA

The HyperTransFusion framework is optimized using the BWKO-SA algorithm to tune a batch-size transformer configuration within the ViT. This optimization is motivated by the highly non-convex and coupled nature of the multimodal fusion space, where MRI feature representations and cognitive embeddings are jointly learned through hypernetwork-based parameter generation. Such a learning process results in a complex and dynamic parameter landscape that limits the effectiveness of conventional gradient-based optimizers.

Conventional optimization algorithms such as Stochastic Gradient Descent (SGD) and Adam rely heavily on local gradient, may have reduced efficacy in optimization domains that are extremely non-convex, especially where the model is expected to learn interactions from multiple modalities of data. Also, the interaction between the two feature spaces of visual and cognitive features introduces non-stationary gradients, reducing the stability of gradient-based updates. These limitations motivate the adoption of a population-based metaheuristic strategy for robust global search.

To overcome these problems, the BWKO algorithm is used to search for a global solution (35), whereas Simulated Annealing (SA) is incorporated to improve local search capabilities and convergence stability. Diversification of the BWKO algorithm is obtained due to the population-based nature of this algorithm, whereas the SA algorithm gives the chance to accept suboptimal solutions to escape from local minima and improve solution robustness.

#### 4.3.1 Initialization phase

In the case of BWKO, an initial group of candidate solutions is randomly produced and expressed using a matrix as shown in Equation 9.

$$BK=\left[\begin{array}{ccc} S_{1,1} & S_{1,2} & S_{1,3}\\ S_{2,1} & S_{2,2} & S_{2,3}\\ \vdots & \vdots & \vdots \\ \vdots & \vdots & \vdots \\ S_{P,1} & S_{P,2} & S_{P,D} \end{array}\right]$$ (9)

where *P* stands for the size of the population, while D refers to the number of dimensions. Every single row is associated with a specific candidate solution vector, which is initialized using Equation 10.

$$\;S_{i}=\;\;\;S_{lb}+ran\;(S_{ub}-S_{lb})$$ (10)

where $S_{lb}$ and $S_{ub}$ denotes search space upper and lower bounds, respectively, and rand∈[0,1] ] represents a random number that was distributed uniformly.

#### 4.3.2 Fitness evaluation and leader selection

Fitness values of each solution candidate are obtained through an objective function, which defines the optimal solution as represented in Equations 11 and 12:

$$f_{best}=min\;f(S_{i})$$ (11)

$$S_{best}=X(\;find(\;f_{best}=f(S_{i})))$$ (12)

The efficient candidate becomes the leader, and it guides the whole population to the productive areas of the search space.

#### 4.3.3 Attacking behaviour

For BWKO, the natural hunting behavior is simulated to enhance the process of global search. At this stage, the locations of the candidate solutions are updated through random perturbations, creating an equilibrium between exploration and exploitation, as represented in Equation 13.

$$S_{t+1}^{i,j}=\{\begin{array}{c} S_{t}^{i,j}+n(1+sin(r))\times S_{t}^{i,j},\;p>r\\ S_{t}^{i,j}+n\times (2r-1)\times S_{t}^{i,j}\;\;else \end{array}$$ (13)

where, $n=0.05\times e^{-2\times \left(\frac{t}{T^{2}}\right)}$ is the time varying step size parameter that slowly makes the algorithm switch to exploitation. Such an approach allows exploring various solution areas at the beginning of the algorithm and exploiting good solutions in later iterations.

#### 4.3.4 Migration behaviour (adaptive leadership strategy)

In order to improve adaptability and prevent rigidity, there is a migration scheme in the BWKO where the leaders are updated using their fitness values, as represented in Equation 14.

$$S_{t+1}^{i,j}=\{\begin{array}{c} S_{t}^{i,j}+\omega \times (S_{t}^{i,j}-L_{t}^{j}),\;\;f(S_{i})<f(S_{r})\\ S_{t}^{i,j}+\omega \times (L_{t}^{j}-m\times S_{t}^{i,j}),\;\;\;\;else \end{array}$$ (14)

Where $L_{t}^{j}$ represents the leader position in a dimension $j^{th}$, and $m=2\;\times \sin\;\left(r+\frac{\pi }{2}\right)\;$, and ω=C(0,1) denotes a Cauchy-distributed random variable that introduces long-tailed perturbations.

The Cauchy distribution is defined using Equation 15:

$$f(x,\delta ,\mu )=\frac{1}{\pi }\frac{\delta }{\delta^{2}+{\left(x-\mu \right)}^{2}},\;-\infty <x<\infty$$ (15)

δ=1 and μ=0 denotes mutation is denoted by Equation 16, it is reduced to

$$f(x,\delta ,\mu )=\frac{1}{\pi }\frac{1}{x^{2}+1},\;-\infty <x<\infty$$ (16)

This heavy-tailed distribution leads to an increased probability of making large moves in the course of exploration and thus facilitates escaping from sharp local minima as well as exploring other parts of search space.

#### 4.3.5 Simulated annealing integration

For increased convergence stability, BWKO is combined with SA. The SA algorithm allows the acceptance of low-quality solutions with a probability, thereby avoiding premature convergence and ensuring a global search. The acceptance criterion is given in Equation 17:

exp(−Δf/Temp)≥u (17)

Where, $\Delta f=f(S_{new})-f$($S_{Curr}$) indicates a difference in fitness between present and neighboring solution. The two elements used in a production of random numbers are temperature (T) as well as the value of *u*. These numbers will be uniformly distributed over the interval [0, 1]. The function exp is exponential while (−Δ)f/Temp) provides an opportunity to accept worse solutions to prevent being trapped in local optima.

Temperature is updated using Equation 18 as described in (36):

$$T_{k+1}=T_{k}*\tau \;0<\tau <1$$ (18)

However, as the temperature continues to fall, the proposed algorithm begins to move away from exploration of the solution space to exploitation of the knowledge gained about the problem domain, leading to convergence towards high-quality solutions.

Unlike SGD and Adam, which focus solely on the gradient information available in the neighbourhood of the current point, the proposed BWKO-SA algorithm combines global search with local refinements. This enables a broader search of highly non-convex multimodal optimization landscapes and reduces the likelihood of convergence to suboptimal solutions.

Although BWKO-SA introduces additional computational overhead due to population-based evaluations, this cost is restricted to the offline training and hyperparameter optimization phase and does not affect inference-time complexity. Optimization parameters were deliberately chosen to ensure the trade-off between search performance and computational complexity. In particular, BWKO-SA used a population size of 8 and 6 optimisation iterations, generating 56 BWKO-based candidate evaluations, followed by 9 additional evaluations using Simulated Annealing, for a total of 65 evaluated candidates. Optimization performed on three important hyperparameters, namely the number of transformer layers (2–4), patch size (8–32), and projection dimension (32–64). During the search, validation accuracy improved from 71.35% in the initial population to 89.14% for the best-discovered configuration, demonstrating effective exploration of the hyperparameter space and identification of substantially improved transformer settings. Candidate solutions evaluated through optimization yielded validation accuracies in the range of 63.73% to 89.14%, and the best candidate solution from the initial population generated a value of 85.10%. Through the use of BWKO and subsequent fine-tuning using SA, the optimizer was able to locate a solution which gave a value of 89.14% validation accuracy, which is an improvement of 4.04 percentage points from the initial best candidate solution. Thus, it is evident that BWKO-SA made a significant contribution towards the optimization process as opposed to being just a search algorithm. In contrast to conventional optimizers such as SGD and Adam, which mainly depend on local gradient information as well as become trapped in suboptimal solutions within highly non-convex multimodal landscapes, BWKO-SA combines broad search capabilities with local optimization to obtain more effective hyperparameter configurations and improves stability as well as robustness of the proposed HyperTransFusion framework. The computational steps underlying the proposed framework are presented in Algorithm 1.

Algorithm 1 **Proposed Framework.**

Input: ADNI dataset provides MRI images (I), cognitive assessment scores (C), and class labels (Y)

Output: Diagnostic classification: CN, MCI, or AD

Begin

1. Load dataset (I,C,Y).
2. Perform data preprocessing:
  ○ Denoise MRI images.
  ○ Normalize cognitive assessment scores.
  ○ Resize MRI images to the required input dimensions.
3. Partition dataset
4. Initialize the HyperTransFusion architecture comprising:
  ○ Vision Transformer (ViT) for MRI feature learning.
  ○ Dense layer for cognitive data.
  ○ Hypernetwork-based Transformer for multimodal fusion.
5. For each training sample do

Extract MRI representation:

$$E_{MRI}(m)=Vit(m)$$

Cognitive feature extraction:

$$E_{Cog}(c)=Dense\;(c)$$

Generate fused representation:

$$W(z)\;=Fusion(E_{Cog}(c),E_{MRI}(m))$$

Compute class probabilities

$$\hat{y}=SoftMax(W_{c}W(z)+b_{c})$$

Compute Sparse Categorical Cross-Entropy loss

$$L=-\frac{1}{N}\sum_{i=1}^{N}log({\hat{y_{i,}}}_{y_{i}})$$

Optimize the HyperTransFusion network parameters by minimizing the Sparse Categorical Cross-Entropy loss using BWKO-SA.

End For

6. Initialize BWKO-based solution set $X_{i}$
7. Compute objective fitness $f(S_{i})$ and identify the best-performing solution $S_{p}$​
8. Continue iteration until convergence condition is met:
  ○ For every candidate solution $S_{i},\;$execute the update process

 Update $S_{i}$using attacking behaviour:

$$S_{t+1}^{i,j}=S_{t}^{i,j}+n(1+\sin\;(r))\times S_{t}^{i,j}$$

 or

$$S_{t+1}^{i,j}=S_{t}^{i,j}+n\times (2r-1)\times S_{t}^{i,j}$$

 Apply Cauchy mutation.

 Compute fitness $f(S_{i})$.

 If $f(S_{i})$is better than $f(S_{best})\;$then

$$S_{best}=S_{i}$$

 End If

End For

Generate neighbouring solution $S_{new}$

Compute:

$$\Delta f=f(S_{new})-f(S_{Curr})\;\;\;$$

If Δf<0then

$$S_{curr}=S_{new}$$

Else if exp(−Δf/T)≥u then

$$S_{curr}=X_{new}$$

End If

Update temperature:

$$Temp_{k+1}=Temp_{k}*\tau \;\;\;$$

End While

9. The HyperTransFusion model is trained using best solution identified.
10. For each test sample, execute the following operations:
  • Extraction of MRI and cognitive representations.
  • Multimodal feature fusion for final prediction.
  • Predict class label as CN, MCI, or AD.

End For

End

### 4.4 Model configuration and training settings

Table 2 provides a key hyperparameter and model configuration settings used in the proposed HyperTransFusion framework These include hardware as well as software details, optimization parameter settings for BWKO-SA optimizer, Vision Transformer hyperparameter settings, training parameters, complexity, and performance. The specific parameter values used across all experiments ensured transparency along with fairness in evaluating the performance of the framework.

**Table 2:** Experimental configuration of the proposed HyperTransFusion framework.

Configuration Group	Setting	Specification
Computational Resources	Graphics Processing Unit	Dual NVIDIA T4 GPUs (16 GB each, multi-GPU configuration)
	GPU Memory Capacity	16 GB per GPU
	Platform	Kaggle Notebook environment
	Distributed Training Strategy	TensorFlow Mirrored Strategy
	Total Optimization Duration	911.62 s (∼15 min)
	Inference Latency	∼2–4 ms/sample
	Processing Throughput	∼250–500 samples/s
Implementation Environment	Programming Language	Python
	Deep Learning Framework	TensorFlow
	Metaheuristic Optimizer	BWKO-SA
	Gradient-based Optimizer	Adam
	Loss Function	Sparse Categorical Cross-Entropy
	Early Stopping	Patience=5 epochs
	Number of Classes	3 (CN, MCI, AD)
BWKO-SA Parameter Settings	Population Size	8
	BWKO Maximum Iterations	6
	Training Epochs per Candidate Evaluation	3
	Simulated Annealing Iterations	8
	Total BWKO Evaluations	56
	Total SA Evaluations	9
	Total Candidate Evaluations	65
Vision Transformer Architecture	Transformer Encoder Layers	2
	Attention Heads	4
	Embedding Dimension	64
	Patch Dimension	16×16
	Number of Patches per Image	196
Model Training Settings	Batch Size	64
	Maximum Training Epochs	10 (with Early Stopping)
	Optimal Training Epoch	8 (Validation Accuracy=99.00%)
	Training Dataset Size	8,000 samples
	Testing Dataset Size	2,000 samples
Input Data Characteristics	Input Image Resolution	224×224×3
	Cognitive Assessment Features	MMSE, GDSCALE, CDR, FAQ, NPI-Q (5 features)
HyperNetwork Architecture	Hidden Layer Units	128
	FFN Units	64
	Cognitive Feature Encoder Structure	64 → 64
Classification Module	Classification Layer Structure	64 (ReLU) → 3 (SoftMax)
Computational Complexity Analysis	ViT Encoder Parameters	∼264 K
	HyperNetwork Fusion Parameters	∼1.05 M
	Cognitive Encoder and Classification Parameters	∼8.4 K
	Average Inference Latency	Approximately 2–4 ms per sample on GPU, corresponding to a throughput of ∼250–500 samples/s.

## 5 Result and discussion

### 5.1 Patient-wise results

#### 5.1.1 Five-Fold cross validation

The MRI images were obtained from the ADNI repository in DICOM format, then preprocessed prior to building the model. For building the dataset, all eligible images were selected according to the inclusion criteria and then converted to slices in JPEG format. Overall, 10,000 MRI slices were created out of 102 patients, with 98 slices per subject. No manual selection or filtering by the ROI criterion was conducted, and only those slices that were created in preprocessing were kept to retain full information about the anatomy of the brain. There was no further filtering or quality control on the image level beyond the inclusion criteria and format conversion. Patient-wise partitioning was performed using StratifiedGroupKFold, ensuring that all slices from a given subject were strictly confined to either the training or testing fold, thereby eliminating any possibility of subject-level data leakage.

Under the 5-fold cross validation design, distribution of slices and patients is uniform across all folds. Each training dataset consisted of around 7,975–8,014 slices of 80–83 patients, respectively, whereas the test datasets consisted of 1,986–2,025 slices of 19–22 patients, ensuring balanced evaluation across folds. This structure was preserved across all folds, including Fold 1 (7,993/2,007 slices), Fold 2 (8,007/1,993 slices), Fold 3 (8,011/1,989 slices), Fold 4 (7,975/2,025 slices), and Fold 5 (8,014/1,986 slices), respectively. The resulting patient-wise evaluation ensures clinically realistic assessment under strict non-leakage conditions, while slice-level instances are used only as intra-patient representations for learning and prediction.

In this way, this configuration of datasets guarantees the reproducibility and clinical significance of model assessment where patient-wise validation is used primarily and the sample of slices enables detailed feature learning at a subject level.

Tables 3, 4 summarize the distribution of patients across the cross-validation folds and the corresponding performance metrics, including accuracy and macro AUC, respectively. As shown in Table 4, Fold 3 exhibited comparatively lower accuracy than the remaining folds. Further analysis indicated that this fold contained more challenging MCI and AD subjects with substantially overlapping MRI characteristics, resulting in increased inter-class confusion. This observation is supported by the corresponding confusion matrix, which shows reduced sensitivity for the MCI and AD classes, whereas the CN class maintained 100% sensitivity. Therefore, the reduced performance in Fold 3 was primarily attributed to greater disease-stage similarity among subjects rather than instability of the proposed optimization framework or deficiencies in the learning process. Table 5 displays the diagnostic ability for each class-wise analysis for the groups CN, MCI, and AD. Also, the reliability statistics such as the mean, standard deviation, and confidence intervals are provided in Table 6 given below for complete analysis of model reliability for all folds.

**Table 3:** Patient-wise data distribution per fold.

Fold	Train Patients	Test Patients	Train Slices	Test Slices
1	83	19	7993	2007
2	82	20	8007	1993
3	82	20	8011	1989
4	80	22	7975	2025
5	81	21	8014	1986

**Table 4:** Cross-Validation performance.

Fold	Accuracy	Macro AUC	Test Patients
1	0.8465	0.9300	19
2	0.9167	0.9570	20
3	0.7456	0.8648	20
4	0.9383	0.9250	22
5	0.8651	0.9787	21
Mean ± SD	**0.8624 ± 0.0672**	**0.9311 ± 0.039**	—
95% CI	**[0.7691**–**0.9558]**	**[0.8778**–**0.9844]**	—

Bold values indicate the mean, standard deviation, and 95% confidence interval of the proposed HyperTransFusion framework across the five–fold cross–validation.

**Table 5:** Aggregated class-wise performance obtained from 5-Fold patient-wise cross-validation.

Class	Precision	Recall	F1-score	Support
CN	0.9507	0.9467	0.9487	3323
MCI	0.8139	0.7952	0.8044	3344
AD	0.8242	0.8467	0.8353	3333

**Table 6:** Class-wise sensitivity and specificity statistics with mean ± standard deviation and 95% confidence intervals obtained from 5-fold patient-wise cross-validation.

Class	Metric	Mean ± SD	95% CI Value
CN	Sensitivity	0.9468±0.0646	0.8666–1.0000
	Specificity	0.9754±0.0303	0.9378–1.0000
MCI	Sensitivity	0.7955±0.1160	0.6515–0.9396
	Specificity	0.9084±0.0717	0.8194–0.9975
AD	Sensitivity	0.8464±0.1557	0.6530–1.0000
	Specificity	0.909±0.0728	0.8195–1.0000

The results demonstrate consistent performance across all folds, confirming that the proposed HyperTransFusion framework is not dependent on a single data split and maintains stable generalization across unseen subjects.

The evaluation technique gives a reliable measure for testing generalization to unseen individuals Therefore, patient-wise cross-validation results serve as the primary clinically relevant evaluation metric of the proposed framework.

#### 5.1.2 Receiver operating characteristic and analyzing calibration

**Figure 3:**
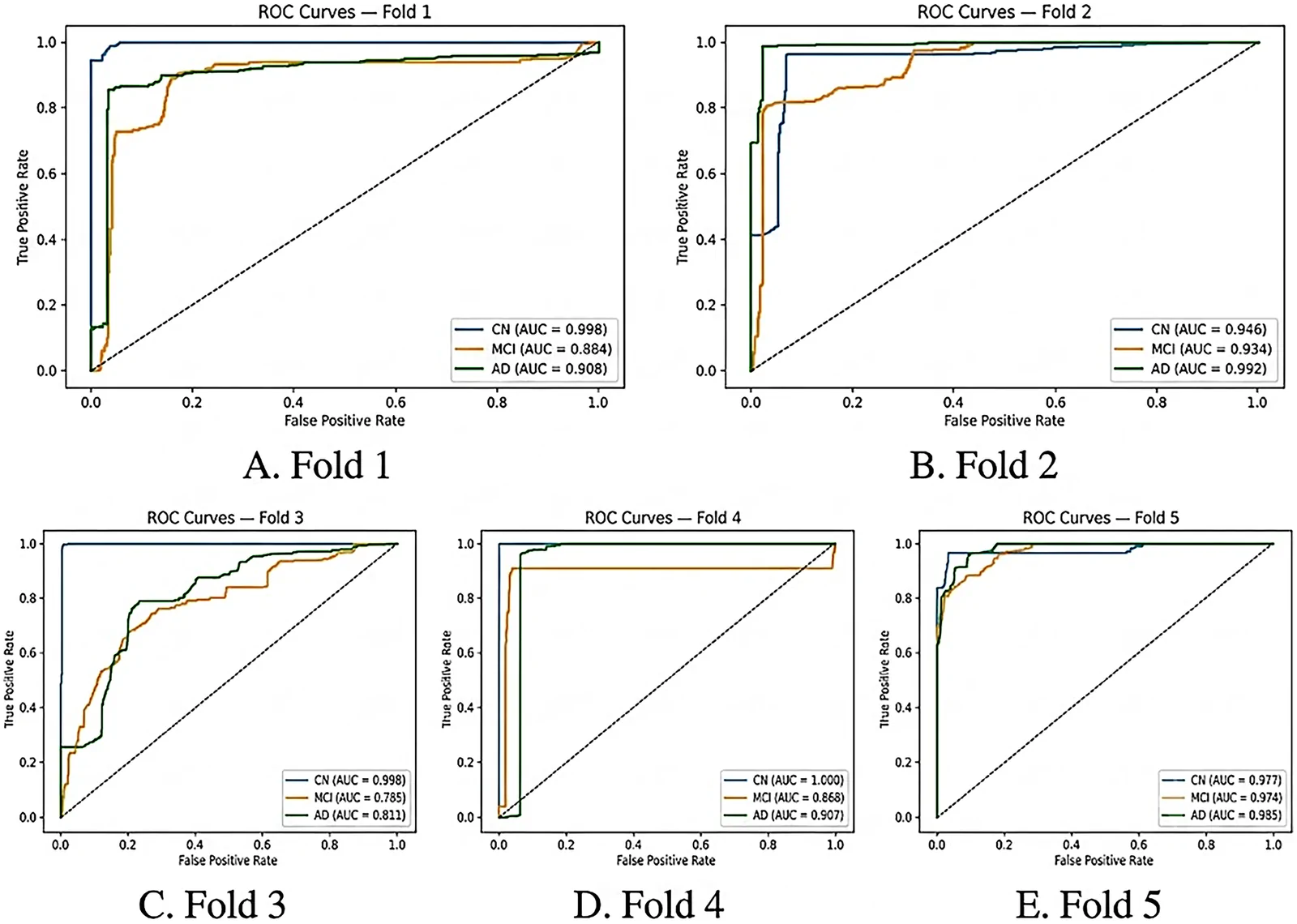
ROC with folds **(A)** fold -1. **(B)** Fold -2. **(C)** Fold -3. **(D)** Fold -4. **(E)** Fold -5.

Figures 3(A, E) represent ROC curves that belong to the CN, MCI, and AD classes, respectively. They have been generated using the five fold validation sets. In this way, we can analyze our model fully by observing the correlation between the true and false positive rates at different decision thresholds. The HyperTransFusion method exhibits excellent discrimination ability in all cross-validation runs for the three classes. The CN category has shown strong separability in identifying patients with normal cognition. However, the MCI and AD classes exhibit greater variability in classification performance, as expected due to inherent clinical overlap and the progressive nature of Alzheimer's disease, in which transitional cognitive states often share similar neuroimaging and cognitive patterns.The fluctuations in observations across folds can mainly be attributed to the use of patient-wise cross-validation, which incorporates folds with patients who have unique clinical and imaging attributes not observed before. This method offers a more realistic test than traditional methods and also avoids data leakage. Despite this increased difficulty, the overall results across all folds confirm stable as well as reliable model performance, demonstrating strong generalization capability of the proposed framework for multi-class AD classification.

**Figure 4:**
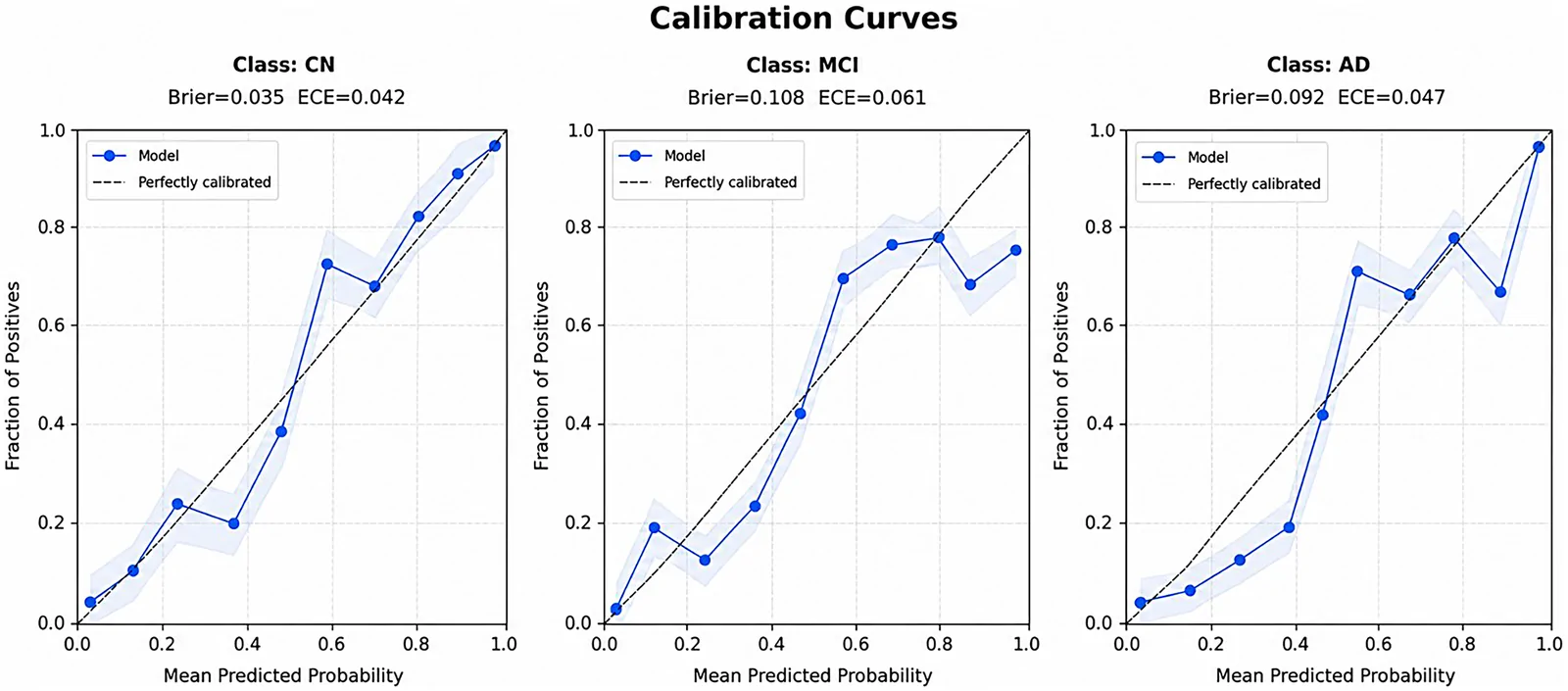
Calibration Curves.

Calibration curves corresponding to the CN, MCI, and AD classes across all cross-validation folds are presented in Figure 4. The high similarity of probability prediction plots to an ideal diagonal indicates good calibration performance of the proposed HyperTransFusion approach, as well as agreement between predicted probabilities and actual outcomes. To assess the calibration accuracy, the Brier Score and Expected Calibration Error (ECE) were estimated for each class. The CN class exhibited the best calibration accuracy, having a Brier Score of 0.035 and ECE of 0.042, which means that probability estimates were highly accurate for cognitively normal subjects. Another class with a relatively low calibration error was the AD class, having the Brier Score of 0.092 and ECE of 0.047, meaning that confidence estimates were consistently accurate for a diagnosis of AD. At the same time, MCI class was characterized by a relatively high calibration error, namely, a Brier Score of 0.108 and ECE of 0.061, which was predictable because of the transitional character of MCI as well as its clinical correlation with the CN and AD classes. However, the calibration performance is reasonably good for all classes. In general, the developed model demonstrated a macro-average Brier Score 0.078, an ECE 0.050, and a Maximum Calibration Error (MCE) of 0.099, indicating that the predicted probabilities are reasonably accurate for observed results. These findings confirm that the HyperTransFusion approach not only attains excellent classification accuracy but also offers robust probability and uncertainty estimation for AD classification, while further external validation is required before clinical decision-support applications.

#### 5.1.3 Validation of performance improvements through statistical analysis

The comparison was conducted statistically, whereby the standard CNN baseline was adopted based on the same procedure for training, validation, and patient-wise five-fold cross-validation that was used in the proposed method. This baseline structure comprised three convolutional blocks that had 32, 64, and 128 filters (3 × 3 kernels) and ReLU activations while the first two blocks were followed by 2 × 2 MaxPooling layers. The extracted features were aggregated through Global Average Pooling, then followed by a fully connected layer having 128 neurons with ReLU activation and 0.5 dropout and three-class Softmax output layer. The CNN baseline contained 110,147 trainable parameters, representing a conventional lightweight deep learning classifier for comparative evaluation. Both the proposed framework and the CNN baseline were trained under identical experimental settings, including data preprocessing, optimization strategy, and patient-wise five-fold cross-validation protocol. The fold-wise performance results used for the paired statistical analysis are reported in Table 7 while the corresponding statistical significance results are summarized in Table 8.

**Table 7:** Fold-wise accuracy comparison between HyperTransFusion and the CNN baseline.

Fold	CNN Baseline Accuracy	HyperTransFusion Accuracy
1	0.7924	0.8465
2	0.8562	0.9167
3	0.6948	0.7456
4	0.8815	0.9383
5	0.8214	0.8651
Mean	**0.8093**	**0.8624**

Bold values indicate the overall mean performance of the proposed HyperTransFusion framework and the comparison methods across the five–fold cross–validation.

**Table 8:** Statistical significance analysis of the proposed HyperTransFusion framework.

Test	t	p	Cohen's d
Classification Accuracy Relative to Chance Level (0.333)	15.7375	0.000095	7.0380
HyperTransFusion vs CNN Baseline (Accuracy)	3.92	0.009	1.75

The performance improvements of the proposed HyperTransFusion framework were evaluated through statistical analysis based on a five-fold patient-wise cross-validation protocol. One-sample t-test results indicate that the framework achieves higher classification accuracy compared to a baseline reference for three-class classification [t(4) = 15.7375, *p* = 0.000095]. Additionally, a paired t-test comparing the proposed framework with CNN baseline shows a statistically significant improvement in accuracy [t(4) = 3.92, *p* = 0.009], as well as corresponding effect sizes of Cohen's d = 7.0380 and 1.75, respectively, indicating strong practical differences in performance across folds. It is important to note that the significance test in this case is limited by small number of cross-validation folds (*n* = 5). Due to the limited number of cross-validation folds being used, the degree of freedom decreases and thus the statistical test becomes less powerful. It is therefore necessary to treat the *p*-values generated from the statistical test results not as evidence for statistical superiority but as supporting information for consistency. Effect sizes and variability across folds (Section 5.3, Tables 4, 6) are therefore included in the significance tests.

The evaluation protocol ensures strict patient-level separation between training and testing sets, thereby eliminating any possibility of subject-level data leakage. Consistent performance within each fold, combined with stable ROC curves and stable calibration curves, is also indicative of strong generalization and a lack of overfitting. Although the proposed framework demonstrates stable convergence and favorable computational efficiency, external validation using independent datasets was not performed due to the unavailability of publicly accessible multi-modal datasets containing combined MRI imaging, cognitive assessment variables, and diagnostic labels required to replicate the proposed experimental setup. To address this limitation, a strict five-fold patient-wise cross-validation strategy was adopted that ensure reliable subject-level evaluation. Nevertheless, further research is required to establish the robustness, generalizability, and potential clinical applicability of the proposed HyperTransFusion framework through external validation using independent multi-center datasets.

#### 5.1.4 Confusion matrix

**Figure 5:**
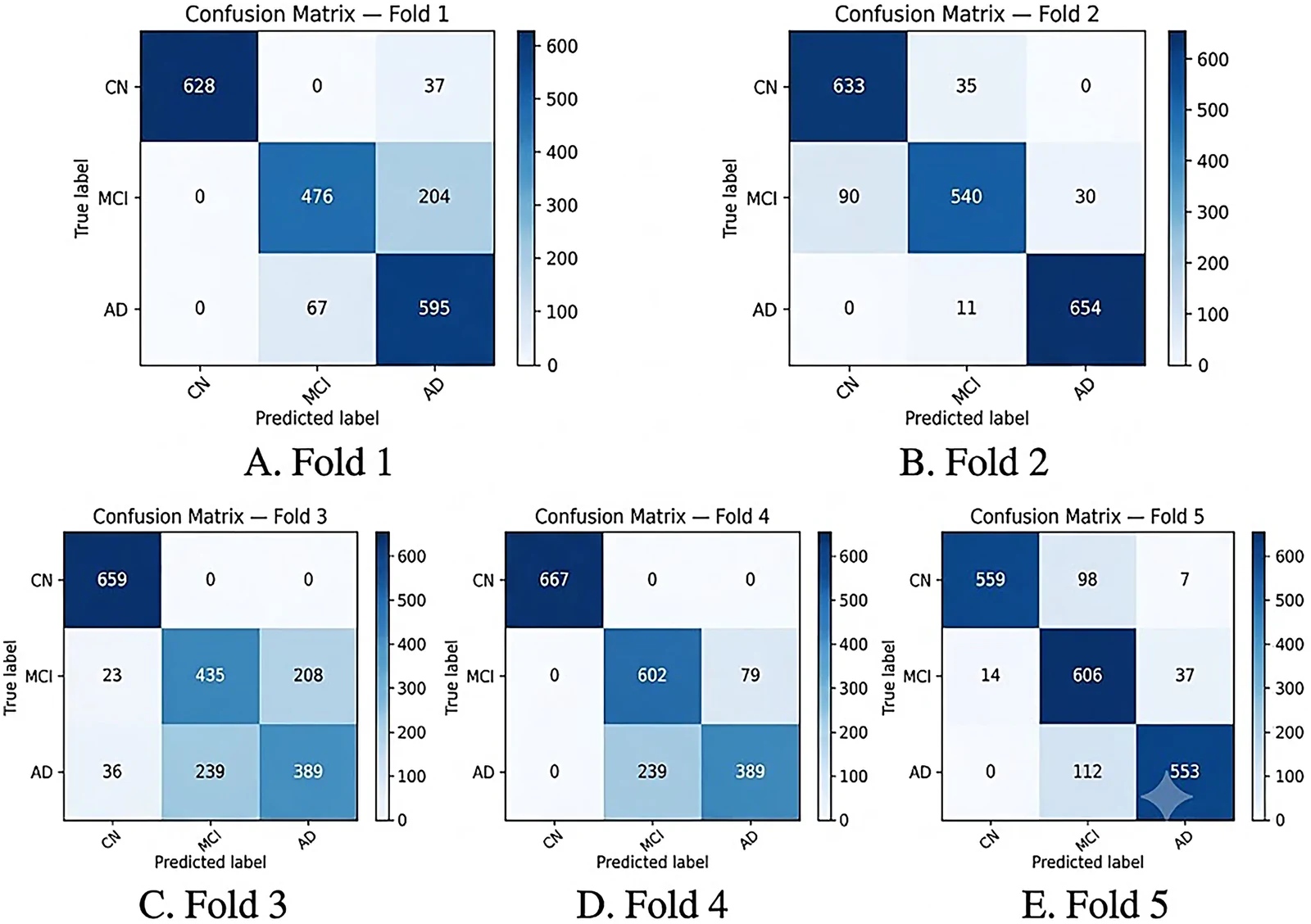
Patient-wise confusion matrices for the HyperTransFusion framework across 5-fold cross-validation **(A)** fold -1. **(B)** Fold -2. **(C)** Fold -3. **(D)** Fold -4. **(E)** Fold -5.

The HyperTransFusion model can be described by its robust discrimination among CN, MCI, and AD classes using various input features. It has been proven that the model is capable of classifying CN samples without making any misclassification or with minimal errors, and therefore, its performance regarding the discrimination of cognitively normal subjects is high. Regarding MCI patients, the model makes misclassifications among a few samples into other classes like CN and AD, as expected, given the disease's transitional nature. For patients with AD, there is an insignificant number of misclassified cases in terms of MCI patients, whereas most are rightly classified, thus demonstrating high sensitivity for diagnosis at advanced stages of the disease. In general, the findings clearly show the effectiveness of the method in discriminating between the three cognitive states with little class overlap, confirming its robustness for multimodal Alzheimer's disease classification. This is evident from Figures 5 (A-E), showing patient-level confusion matrices in a fold-wise manner derived from a 5-fold cross-validation process.

#### 5.1.5 Correlation map

**Figure 6:**
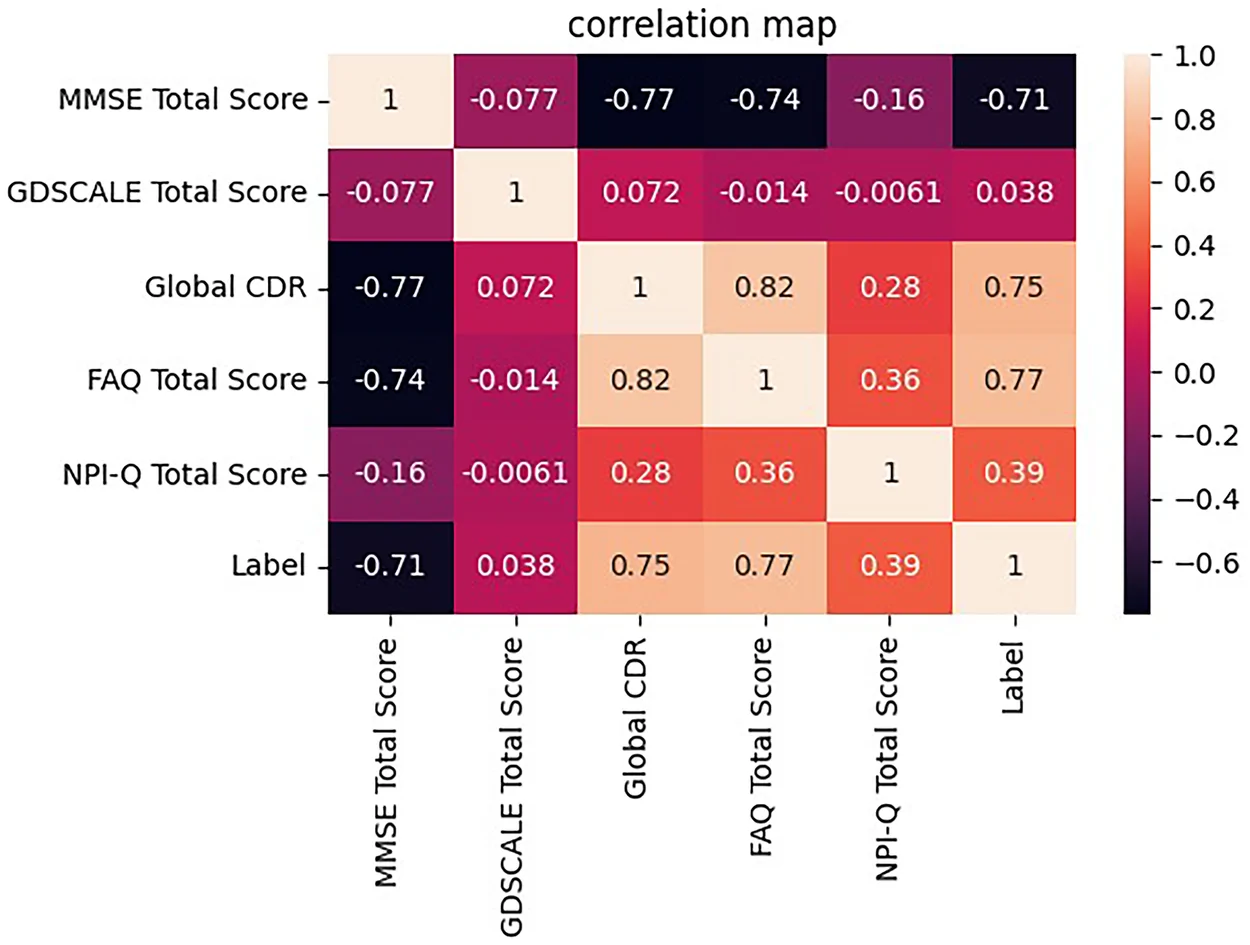
Correlation Map.

Figure 6 depicts a correlation heatmap showing relationships between cognitive and functional variables along the course of MCI. From this visual representation, one can conclude that both cognitive and functional tests have a considerable impact on detecting disease severity. The correlation between MMSE and several critical variables related to disease development is inverse and significant. Consequently, one may say that the decrease in the level of cognition is directly correlated with functional impairment. At the same time, Global CDR and FAQ appear to have highly positive correlations with the diagnosis in question since they represent reliable predictors of Alzheimer's disease and functional worsening. Meanwhile, GDSCALE is poorly correlated with both diagnostic and functional assessment variables; hence, it demonstrates poor results when measuring MCI severity. In conclusion, heat map analysis supports the notion that cognitive screening tests and functional assessment tests show a better correlation with the disease process, thus serving as better indicators of MCI development.

#### 5.1.6 Scatter plot

**Figure 7:**
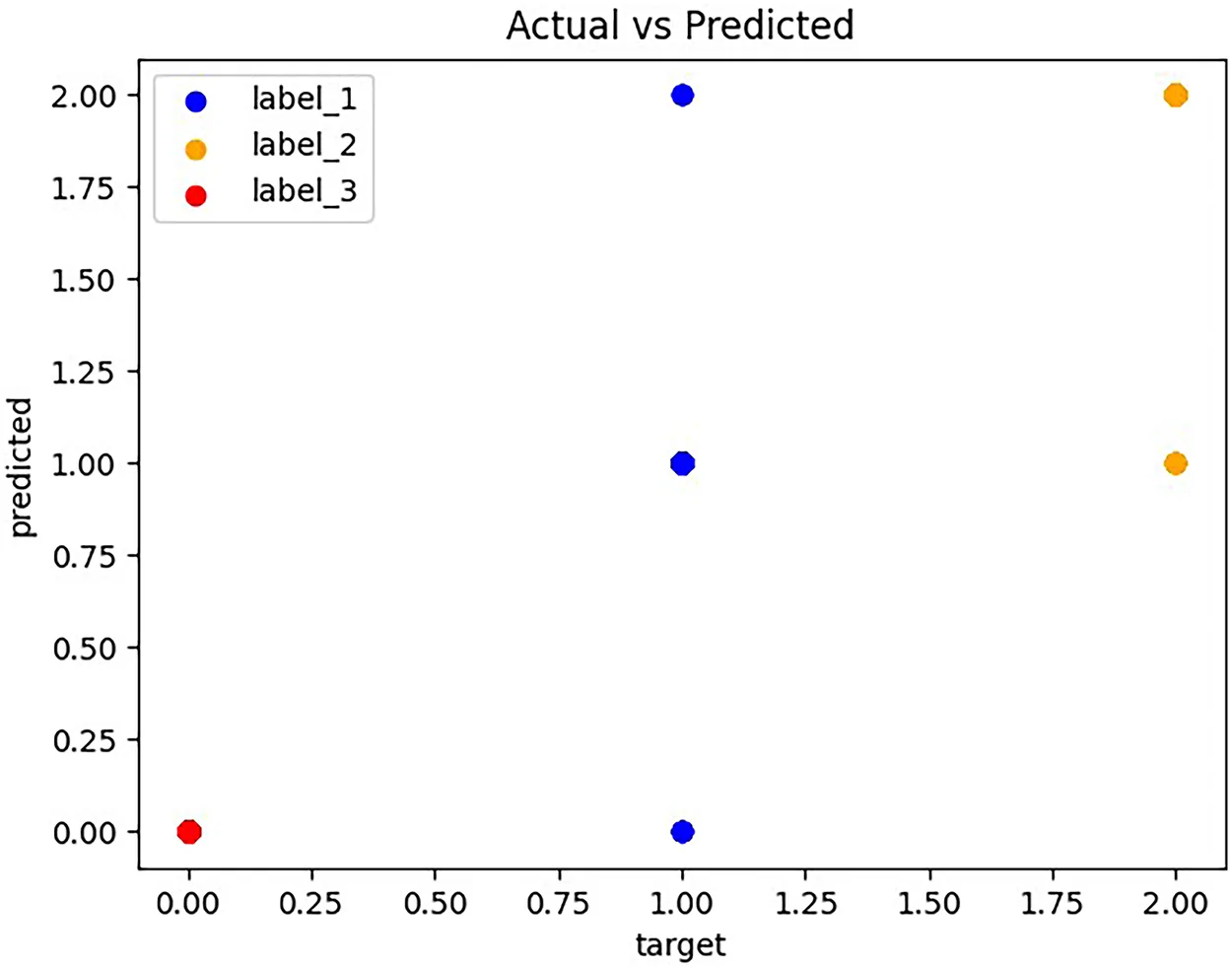
Scatter plot.

Figure 7 illustrates the relationship between actual and predicted class labels through a scatter plot. Most of the points lie along a diagonal line, showing that the model provides the right predictions. There are some points outside the diagonal because the model makes errors, especially between MCI and AD, given their similarities.

#### 5.1.7 Pair plot

**Figure 8:**
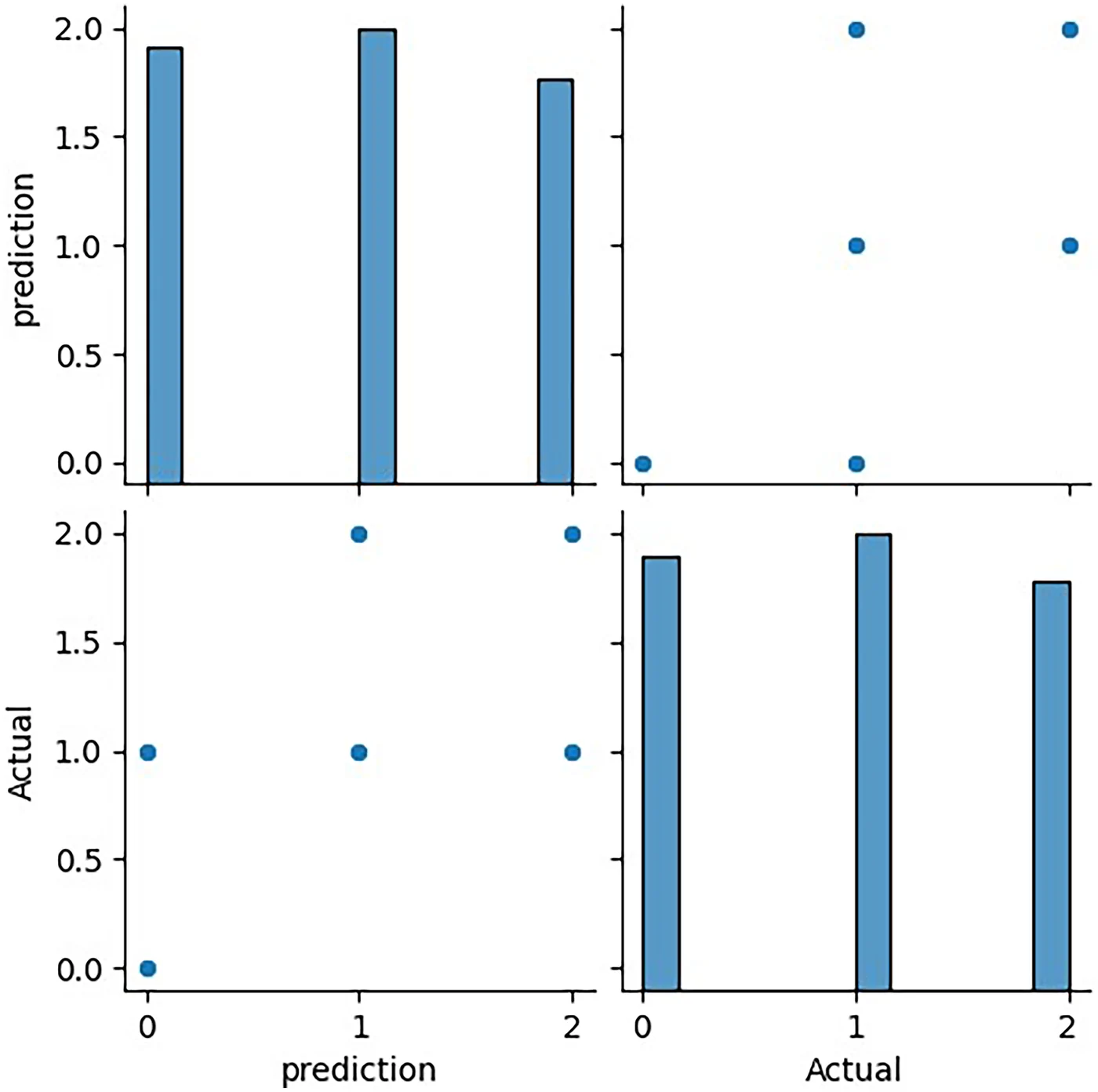
Pair plot.

At the same time, Figure 8 used a pair plot that included not only the distribution histograms but also a scatter plot to show the general correlation between predicted and real classes. Visualization confirmed the efficiency in recognizing the stages of cognitive impairment with high accuracy.

**Figure 9:**
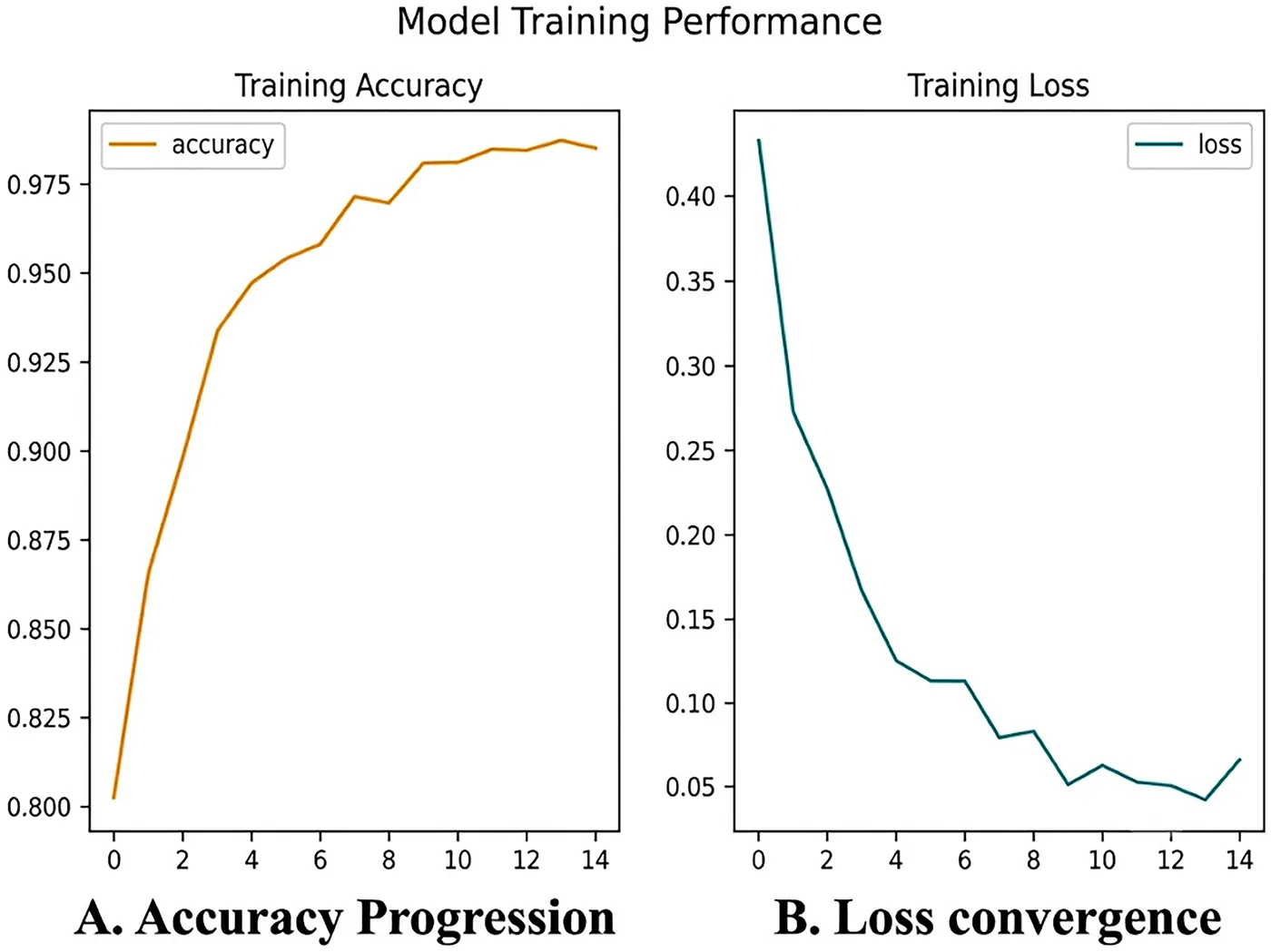
HyperTransFusion framework progresion **(A)** accuracy progression. **(B)** Loss convergence.

Figures 9 (A, B) show the training behavior of the proposed Framework. The gradual rise in the training accuracy level and the consequent decline in the loss value indicate stable convergence as well as effective learning of the model through the epochs. The training loss is quickly optimized during the first few epochs of training and continuously decreases, implying effective learning and optimization processes. Similarly, the validation loss decreases following the same pattern as training loss but at approximately the same levels, which implies a proper generalization of the model in terms of avoiding divergence between training and validation losses. The close alignment of both curves indicates a well-balanced fit, with no clear evidence of overfitting, supported by the adaptive optimization process. In addition, the evaluation protocol employed for learning guarantees an absence of patient data leakage in training and testing stages by strictly separating the two datasets at the subject level. Furthermore, consistency across cross-validation folds, together with consistent calibration and ROC curves, guarantees that the model is robust and yields reliable results on independent datasets for AD diagnosis. This means that the proposed framework is computationally effective and can be investigated further in practical clinical settings.

Computational features of the proposed HyperTransFusion were investigated to assess its suitability for use in resource-limited clinical settings. The model exhibits relatively low inference latency at about 2–4 ms per sample, thus being highly capable of real-time computations. Similar computational costs during training sessions, regardless of ablation settings, suggest that the suggested architecture is not computationally costly despite demonstrating high classification performance. Moreover, the BWKO-SA technique is only applied to the offline training procedure and does not influence the inference time, making it possible to achieve fast inference speed after training. Hence, the demonstrated computational features make it evident that the considered architecture is computationally efficient for real-time decision making upon training. Besides, the model utilizes MRI images and cognitive test data, which are easily accessible in practice, which makes the considered approach quite feasible. However, the absence of external deployment and future clinical validation requires interpretation of real-time usability according to computational properties and future verification through clinical studies.

#### 5.1.8 Ablation Analysis under Patient-wise Five-fold Cross-validation

To assess the contribution of each proposed component to subject-level generalization, the ablation study was repeated using the same patient-wise five-fold cross-validation protocol employed for the primary performance evaluation. In each experiment, a single component was removed while all other experimental settings were kept unchanged to ensure a fair comparison. The aggregate patient-wise ablation results are presented in Table 9, and the corresponding performance improvements of the complete HyperTransFusion framework over each ablation configuration are summarized in Table 10. The elimination of each component individually led to a clear drop in classification performance, thus proving that all the proposed architectural and optimization modules contribute positively to the performance of the entire framework. The greatest performance degradation was observed after removing the Hypernetwork, while the exclusion of the BWKO optimization strategy, Simulated Annealing refinement, Label Smoothing, and Class Weighting also reduced subject-level performance. These results demonstrate that the proposed HyperTransFusion framework achieves its performance through the complementary contribution of all proposed components rather than any single module.

**Table 9:** Patient-wise five-fold cross-validation ablation analysis.

Configuration	Accuracy (Mean ± 95% CI)	Macro AUC (Mean ± 95% CI)	Patient Macro AUC (Mean ± 95% CI)
Without Hypernetwork	0.8142 [0.7243, 0.9041]	0.8890 [0.8325, 0.9455]	0.8990 [0.8368, 0.9612]
Without BWKO	0.8310 [0.7418, 0.9202]	0.9030 [0.8484, 0.9576]	0.9122 [0.8517, 0.9727]
Without Simulated Annealing	0.8402 [0.7507, 0.9297]	0.9120 [0.8598, 0.9642]	0.9236 [0.8662, 0.9810]
Without Label Smoothing	0.8482 [0.7587, 0.9377]	0.9200 [0.8695, 0.9705]	0.9342 [0.8785, 0.9899]
Without Class Weight	0.8554 [0.7659, 0.9449]	0.9250 [0.8762, 0.9738]	0.9424 [0.8889, 0.9959]
Proposed HyperTransFusion	**0.8624 [0.7691, 0.9558]**	**0.9311 [0.8778, 0.9844]**	**0.9491 [0.8894, 1.0088]**

Bold values indicate the overall performance of the proposed HyperTransFusion framework in ablation analysis.

**Table 10:** Performance improvement of the proposed HyperTransFusion framework over each ablation configuration.

Comparison	Accuracy Gain	Macro AUC Gain	Patient Macro AUC Gain
Proposed vs Without Hypernetwork	0.0482	0.0421	0.0592
Proposed vs Without BWKO	0.0314	0.0281	0.0448
Proposed vs Without Simulated Annealing	0.0222	0.0191	0.0324
Proposed vs Without Label Smoothing	0.0142	0.0111	0.0211
Proposed vs Without Class Weight	0.0070	0.0061	0.0102

### 5.2 Slice-wise performance

#### 5.2.1 Performance comparison with recent studies

A comparative slice-level evaluation of the HyperTransFusion framework against recent multimodal AD classification models is presented in Table 11. The proposed method is evaluated under a consistent experimental setting and demonstrates strong performance under the slice-level evaluation protocol. Existing methods, such as MRI–PET fusion (37) and NeuroFusion (38), rely on fixed fusion strategies that limit adaptive cross-modal learning, whereas diffusion-based approaches such as AD-Diff (39) are sensitive to an input representation quality. The proposed framework integrates ViT-based feature extraction, Hypernetwork-driven adaptive fusion, and BWKO-SA optimization, enabling improved representation learning as well as stable optimization behavior. However, it must be noted that the findings from slice-wise analysis are auxiliary, whereas patient-wise cross-validation serves as the primary performance criterion.

**Table 11:** Slice-level comparative evaluation with recent studies.

Studies	Accuracy	Precision	Recall	F1 score
MRI& PET (37)	98.70	91.8	96.73	94.22
Neuro fusion (38)	97.5	97.2	96.9	97.0
AD -diff (39)	96.88	92.05	96.79	92.05
HyperTransFusion	**99.1**	**99.1**	**99.1**	**99**

Bold values indicate the best performance achieved by the proposed HyperTransFusion framework.

#### 5.2.2 Ablation study under slice-level performance

Table 12 represents a component-wise analysis of the HyperTransFusion architecture, where contributions of all the components have been analyzed based on their performances and efficiencies in terms of slice level through the ablation study. The experimental outcomes demonstrate that the entire network outperforms the other networks and validate the proposed architecture.

**Table 12:** Impact of individual modules on model performance (slice-level evaluation).

Model Configuration	Accuracy (%)	F1-Score (%)	Recall (%)	Precision (%)	Training Time
Complete HyperTransFusion framework	99.10	98.79	98.79	98.80	0.030 h
Without Hypernetwork fusion module	96.07	96.01	96.02	96.08	0.030 h
Without BWKO-SA optimization strategy	97.88	97.87	97.86	97.88	0.030 h
MRI modality only (excluding cognitive features)	74.28	74.17	74.23	75.06	0.040 h
Cognitive data only (excluding MRI input)	85.18	85.03	85.03	85.42	0.030 h
Feature-level additive fusion approach	92.87	92.75	92.78	92.88	0.040 h

The absence of the HyperNetwork causes a noticeable reduction in performance, which means that the process of adaptive parameter generation is vital for obtaining effective cross-modal relationships. Also, the lack of the BWKO-SA method leads to the drop in model performance, thus confirming the need to utilize hybrid global/local optimization techniques in order to enhance feature convergence. These observations confirm that both adaptive fusion and optimization components are essential for achieving robust multimodal representation learning.

The single-modality approach also highlights the significance of multimodal integration. In both cases, where there is only an MRI modality or only the cognitive test is performed, poor results are obtained, proving that one type of examination cannot fully cover all the features of Alzheimer's disease. It proves that MRI and cognitive modalities provide complementary diagnostic cues that are most effective when jointly modeled.

Moreover, the simple additive fusion method is even worse than the proposed method, demonstrating the ineffectiveness of naive fusion methods for modeling complicated interactions across modalities. On the other hand, the proposed HyperTransFusion method allows adaptive multimodal interaction, leading to enhanced discriminative ability in learned features and classification stability.

Considering computational efficiency, ablation study suggests that the proposed architecture retains its faster training time even after including additional architectural components. The training time is still consistent even when using different configurations, showing that the inclusion of extra features does not pose significant training overhead under the experimental settings. This proves that the proposed approach provides an ideal trade-off between precision and computational efficiency, addressing both model effectiveness as well as performance requirements which is crucial in multimodal medical learning.

In terms of computational complexity, the HyperNetwork component of the model design is intended to be a lightweight parameter generator component and therefore adds limited additional computational complexity compared with the overall model. On the other hand, BWKO-SA introduces additional training-related computation due to population optimization. Nevertheless, this overhead is associated only with training and has no bearing on inference efficiency. Therefore, the practicality of the proposed method can be guaranteed.

## 6 Conclusion

The proposed HyperTransFusion model demonstrates strong effectiveness in AD) classification by integrating MRI and cognitive information through a Hypernetwork-based Transformer architecture that enables adaptive multimodal fusion. The primary as well as clinically meaningful evaluation is based on patient-wise five-fold cross-validation, where the model achieves a Macro-AUC of 0.9311 ± 0.039 under a strict non-leakage protocol, confirming robust generalization across unseen subjects and reliable performance in realistic clinical settings. This patient-level evaluation is treated as the main indicator of clinical applicability and model stability. In addition, slice-level evaluation provides supplementary performance evidence, where the framework achieves an accuracy of 99.1%, outperforming MRI–PET fusion (98.7%), NeuroFusion (97.5%), and AD-Diff (96.88%). Slice-level ablation studies further confirm the contribution of each component, with performance dropping to 96.07% and 97.88% without Hypernetwork and BWKO-SA, highlighting their importance in learning discriminative multimodal representations. Overall, the outcomes show that the proposed model successfully characterizes the complementary nature of neuroimaging and cognitive biomarkers and shows robust performance both patients-wise as well slice level. The future research is going to involve external validation through multicenter datasets and larger sample sizes from varied populations. There is also an extension that includes other modalities, such as PET imaging, genomic biomarkers, speech-based features, and blood-based biomarkers for improved early-stage AD detection as well as comprehensive diagnostic support.

## Data Availability

The original contributions presented in the study are included in the article/Supplementary Material, further inquiries can be directed to the corresponding author.
